# Long Intergenic Non-Coding RNAs in the Mammary Parenchyma and Fat Pad of Pre-Weaning Heifer Calves: Identification and Functional Analysis

**DOI:** 10.3390/ani11051268

**Published:** 2021-04-28

**Authors:** Shengchao Zhang, Sibtain Ahmad, Yuxia Zhang, Guohua Hua, Jianming Yi

**Affiliations:** 1 Key Laboratory of Agricultural Animal Genetic, Breeding, and Reproduction for Ministry of Education, College of Animal Science, Huazhong Agricultural University, Wuhan 430070, China; sczhang2021@163.com (S.Z.); yuxiazhang2021@163.com (Y.Z.); 2 Faisalabad-Sub Campus Depalpur, University of Agriculture, Okara 56150, Pakistan; dr_sibtainhmd6@uaf.edu.pk; 3 National Center for International Research on Animal Genetics, Breeding and Reproduction (NCIRAGBR), Huazhong Agricultural University, Wuhan 430070, China

**Keywords:** mammary gland development, pre-weaning stage, lincRNA, functional analysis

## Abstract

**Simple Summary:**

The development of mammary gland is directly related to the productivity of dairy animals. Some studies showed that feeding the enhanced plane of nutrition at pre-weaning stage are advantageous to the development of mammary gland. However, regulators which are involved in this biological process remain largely unknown. In this work, we have identified some long intergenic non-coding RNAs (lincRNAs) in mammary parenchyma (PAR) and mammary fat pad (MFP) of heifer calves under different levels of nutrition at pre-weaning stage by using the published RNA-seq database. Furthermore, those putative lincRNAs, which were highly correlated with these key protein-coding genes in mammary gland development, were highlighted. Our results not only confirmed the advantages of feeding calves with enhanced feeding plane in pre-weaning stage, but also provided fundamental base for further research on the biological processes of mammary gland development.

**Abstract:**

Enhanced plane of nutrition at pre-weaning stage can promote the development of mammary gland especially heifer calves. Although several genes are involved in this process, long intergenic non-coding RNAs (lincRNAs) are regarded as key regulators in the regulated network and are still largely unknown. We identified and characterized 534 putative lincRNAs based on the published RNA-seq data, including heifer calves in two groups: fed enhanced milk replacer (EH, 1.13 kg/day, including 28% crude protein, 25% fat) group and fed restricted milk replacer (R, 0.45 kg/day, including 20% crude protein, 20% fat) group. Sub-samples from the mammary parenchyma (PAR) and mammary fat pad (MFP) were harvested from heifer calves. According to the information of these lincRNAs’ quantitative trait loci (QTLs), the neighboring and co-expression genes were used to predict their function. By comparing EH vs R, 79 lincRNAs (61 upregulated, 18 downregulated) and 86 lincRNAs (54 upregulated, 32 downregulated) were differentially expressed in MFP and PAR, respectively. In MFP, some differentially expressed lincRNAs (DELs) are involved in lipid metabolism pathways, while, in PAR, among of DELs are involved in cell proliferation pathways. Taken together, this study explored the potential regulatory mechanism of lincRNAs in the mammary gland development of calves under different planes of nutrition.

## 1. Introduction

The mammary gland is a complex organ, distinguishing mammals from other animal species, undergoing through a series of developmental changes at different physiological stages starting from embryo to pubertal stage following reproductive stage, also known as mammogenesis. It is composed of different types of cells like parenchyma cells, mammary fat pad (MFP), fibroblasts and vascular endothelial cells [1]. Parenchyma (PAR) is the key tissue in synthesis and secretion of milk, while, the mammary fat pad (MFP) tissue is used to provide the protection and support for PAR [2]. MFP is necessary for development of the secretory epithelium and provides signals that mediate ductal morphogenesis and potentially alveolar differentiation. Moreover, these MFPs are susceptible to dietary changes [3]. Although, mammary gland ducts’ elongation and branching mainly occur in the pubertal period. But, the pre-weaning stage of mammary gland played an important role in heifer’s future milk yield [4,5]. Previous studies showed that the EH and R nutritional levels have different impacts on the mammary gland mass and composition of PAR and MFP in the pre-weaning stage. A number of genes have been identified that are involved in mammary gland development under different dietary planes, like, EGF, FGF2, IGF-1, etc. [6]. Still, there is a dire need to identify novel genes and their interaction in different tissues of the mammary gland.

Long non-coding RNAs (lncRNAs) have been defined as transcripts of length ≥200 nt that lacks protein-coding potential [7]. According to the genomic location and context, lncRNAs are divided into five classes, including intergenic, sense, antisense, intronic and bidirectional non-coding RNAs and vast majority is long intergenic non-coding RNAs (lincRNAs) [8,9]. LincRNAs have diverse different features form messenger RNA (mRNA) and exercise functions such as chromatin modifications and transcriptional regulation in nucleus, and also implied in post-transcriptional regulation in cytoplasm [10]. Besides, some of the lincRNAs have been confirmed to be the key regulators and biomarkers in the development of mammary gland, like, nuclear-enriched abundant transcript 1 (NEAT1), pregnancy induced noncoding RNA (PINC) and zinc finger NFX1-Type containing 1 (znfx1) antisense RNA 1(ZFAS1), etc. [11,12,13]. However, those lincRNAs, which might be involved in mammary development under different nutrient supply in pre-weaning period of Holstein calves are yet to be known [14,15,16,17].

In this study, we identified the lincRNAs in MFP and PAR of pre-weaning Holstein heifers, under enhanced (EH) and restricted (R) plane of nutrition [18]. A total of 534 transcripts originating from 434 gene loci were identified as putative lincRNAs. The function of these putative lincRNAs’ was predicted by analyzing neighboring genes and significantly correlated differentially expressed genes (DEGs) in MFP or PAR under EH and R nutrition supply. The present study broadens the knowledge of lincRNA annotation in bovine as well as facilitates future research about the mammary gland development.

## 2. Materials and Methods

### 2.1. Experiment Design and Library Construction

The experiment was previously published by Vailati-Riboni et al. [18]. Briefly, 12 Holstein heifer calves (6.0 ± 2 d old, 39.0 ± 4.4 kg) were divided into two groups under the same forage and feeding management conditions. Feeding was started at the end of 4th week and both of treatments were reduced to 50% at 8th week, to induce weaning. During the trials, both milk replacers were fed in two equal portions twice daily at 06:00 and 17:00 h and calves were provided with drinking water supply. Total RNAs was extracted from the MFP and PAR after the calves were euthanized and their whole mammary glands were removed and dissected. The RNA-seq cDNA libraries were constructed using the Illumina TruSeq Stranded mRNA Sample Prep kit. The single-end read library construction following the manufacturer’s instructions with mRNA enrichment.

### 2.2. Databases

A total of 22 single-read RNA-seq data were downloaded from NCBI Sequence Read Archive (SRA) database. The Bos taurus UMD3.1.1 reference genome FASTA file and the Gene Transfer Format (GTF) file were downloaded from the ensembl website (ftp://ftp.ensembl.org/pub/release-98/fasta/bos_taurus/dna/ (accessed on 1 June 2014)) and (ftp://ftp.ensembl.org/pub/release-98/gtf/bos_taurus/ (accessed on 1 June 2014)). The UniRef90 (UniProt Reference Clusters) database was downloaded from the UniProt website (http://www.ebi.ac.uk/uniprot/database/download.html (accessed on 1 January 2019)). Moreover, non-redundant reference sequence (RefSeq) NR data was downloaded from (ftp://ftp.ncbi.nih.gov/blast/db/ (accessed on 1 January 2016)). The *Homo sapiens* and *Mus musculus* over.chain file (converting genome coordinates intermediate files) downloaded from (https://hgdownload.soe.ucsc.edu/goldenPath/bosTau8/liftOver/ (accessed on 1 October 2014)). The bed file downloaded from (http://asia.ensembl.org/biomart/martview/ (accessed on 1 June 2014)).

### 2.3. Alignment and Assembly of RNA-Seq Data 

Reads were aligned to *Bos taurus* reference genome (UMD3.1.1) by Hisat2 (version2.1.0, Iowa State University, Ames, IA, USA) with default parameters [19]. Mapped reads were assembled and 22 assembled transcripts files (GTF format) of four groups were then merged into a non-redundant transcriptome by StringTie (version 1.3.5, Johns Hopkins University, Baltimore, MD, USA) [20].

### 2.4. Identification and Characterization of Putative LincRNAs

**Figure 1:**
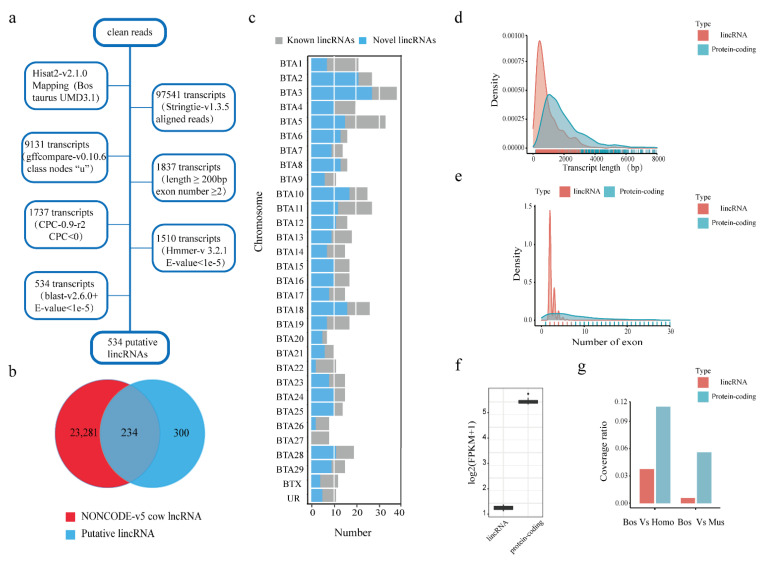
Identification and characterization analysis of the putative long intergenic non-coding RNAs (lincRNAs). (**a**) Pipeline for the identification of putative lincRNAs. (**b**) Venn diagram of known and novel lincRNAs. (**c**) *Bos taurus* chromosome distribution of putative lincRNAs. Blue: novel lincRNAs, gray: known lincRNAs. (UR: unknown region, included NKLS02001272.1, NKLS02002063.1, NKLS02002207.1, NKLS02002210.1, NKLS02001064.1, NKLS02001662.1, NKLS02002183.1). (**d**) Transcript length of putative lincRNAs and protein-coding gene (X axis represent the range of transcription length (bp); Y axis represent the portion of specific length of transcripts); (**e**) exon numbers of lincRNAs and protein-coding gene (X axis represent the range of exon number; Y axis represent the portion of specific number of exon); (**f**) the expression levels of lincRNAs and protein-coding gene; (**g**) evolutionary conservation between protein-coding genes and putative lincRNAs of *Bos taurus (Bos)* with *Homo sapiens (Homo)* and *Mus musculus (Mus)*.

LincRNAs are the intergenic transcript which have been defined as transcribed non-coding RNAs ≥ 200 nucleotides. Based on this, our pipeline to identify lincRNAs has been shown in (Figure 1a).

We used non-redundant transcriptome to identify lincRNAs: (1) only the “u’’ class as candidate linRNAs by gffcompare (version 0.10.6, Johns Hopkins University, Baltimore, MD, USA), which represented the unknown, intergenic transcripts, were retained [21]; (2) transcripts were selected to do further analysis (having exon ≥ 2 and length ≥ 200 bp); (3) We used coding potential calculation (CPC) tool (version -0.9-r2, Tsinghua University, Beijing, China) to calculate the coding potential of transcripts in both strands, CPC < 0 were retained [22]; (4) we used command “transeq” and “hmmerscan” of HMMER (version 3.2.1, HHMI Janelia Fam Research Campus, Ashburn, VA, USA) tool to translated the retained transcript sequence into six possible protein sequences, which had a significant hit in the Pfam database (E-value < 1 × 10^−5^) were removed [23]; (5) we compared retained transcript sequences with NCBINR and UniRef90 database by BLAST(version v2.6.0 +, National Center for Biotechnology Information, Bethesda, MA, USA), with similarity to known proteins (E-value < 1 × 10^−5^) were removed [24]; (6) those transcripts, having fragments per kilobase of transcript per million mapped reads (FPKM) score more than 0.1 at least one sample were retained. In addition, we found that 234 out of 534 putative lincRNA sequences were highly similar to the known lncRNA by comparing the NONCODE database (http://www.noncode.org (accessed on 1 January 2018)) [25]. Kolomogorv–Smirnov (KS) test was used for the characterization of putative lincRNAs and protein-coding genes. Furthermore, UCSC website liftOver tools (http://genome.ucsc.edu/cgi-bin/hgLiftOver (accessed on 2 July 2015)) and the over.chain file were used to compare evolutionary conservation of putative lincRNAs and protein-coding genes.

### 2.5. Express Analysis mRNA and Putative LincRNAs in MFP and PAR

Gene expression levels were estimated based on read counts through feature count (version 1.6.4) software [26]. R package DEseq2 was used to conduct differential expression tests between the two groups. And it was considered differentially expressed, if the value of |log_2_(Fold-change)| ≥ 1 and padj ≤ 0.05 [27]. In addition, unpaired *t*-test was used to identify differentially expressed lincRNAs (DELs).

### 2.6. Neighboring Gene Identification and Correlation Analysis of DELs of MFP and PAR

We identified the neighboring gene (<100 kb) of putative lincRNAs by Bedtools (version 2.29.0, University of Virginia School of Medicine, Charlottesville, VA, USA) [28]. Pearson correlation coefficient as applied to calculate the correlation between DELs and neighboring gene or DEGs. In addition, only putative lincRNAs’ function was inferred according to the pathway analysis result of the genes, which were adjacent to these DELs or significantly correlated with DELs. 

### 2.7. GO Ontology and Pathway Analysis

In order to predict these putative lincRNAs’ function, the KOBAS (version 3.0) (http://kobas.cbi.pku.edu.cn/kobas3/?t=1 (accessed on 5 July 2011)) was used in order to conduct the pathway analysis [29]. *p*-value of pathway and enrichGO less than 0.05 were considered statistically significant.

### 2.8. Quantification of LincRNAs through QRT-PCR

PCR primers for three randomly selected lincRNAs were designed by the oligo7 program (Supplement Table S1). Total RNA was extracted from each target tissue of bovine by using TRIzol reagent (Invitrogen, California, CA, USA) as per the instruction of manufacturer. The Glyceraldehyde-3-Phosphate Dehydrogenase (GAPDH) served as the endogenous control gene. QRT-PCR was performed with SYBR^®^ premix Ex Taq II (Tli RNaseH Plus) (2×) (TAKARA, Kyoto, Japan) to assess the expression level of these three lincRNAs and the results were calculated using the 2^−ΔΔCt^ [30].

## 3. Results

### 3.1. Identification and Characterization of the Putative LincRNAs

A total of 22 RNA-seq data were collected from Gene Expression Omnibus (GEO) database. These data represent all the transcripts of MFP and PAR tissues from pre-weaning Holstein heifer calves, which were treated with restricted milk replacer (R, 0.45 kg/day, including 20% crude protein, 20% fat), and enhanced milk replacer (EH, 1.13 kg/day, including 28% crude protein, 25% fat) to identify lincRNAs in these tissues related to energy supply [18]. By using HISAT2, approximately 579.8 of 600.1 million clean reads were mapped on the *Bos taurus* reference genome (UMD3.1.1) (Supplement Table S2). Then 97,541 non-redundant transcripts were reconstructed for each sample by merged command of the stringtie software. The results of gffcompare showed that 9131 transcripts were intergenic transcripts. Finally, 534 transcripts originated from 434 gene loci were identified as the putative lincRNA based on their protein coding abilities (Figure 1a) (Supplement Table S3). By comparing with the NONCODEv5_cow database (http://www.noncode.org (accessed on 6 January 2018)), we found that 234 out of 534 transcripts have high similarity with known lincRNAs (Supplement Table S4). The other 300 transcripts were considered as novel lincRNAs (Figure 1b). More than 30 lincRNAs were enriched in BTA 3 (*Bos taurus* autosome 3), followed by BTA 5, 2, 10 and 11 (Figure 1c).

There were many differences between lincRNAs and protein-coding genes, including different exon number, length of transcript, expression level and evolutionary conservation, etc. [31] to characterize putative lincRNAs, these were compared with those protein-coding transcripts, which have been annotated in Ensembl database. The characteristics of protein-coding genes and putative lincRNAs were revealed by Kolomogorv–Smirnov (KS) test. These results showed that the average transcript length of these putative lincRNAs was significantly shorter when compared with the protein-coding genes (mean value 967 bp vs. 2058 bp, respectively; *p*-value < 2.2 × 10^−16^). (Figure 1d). Meanwhile, we found that the average exon number of the putative lincRNAs was less than protein-coding genes (mean value 2.4 vs. 9, respectively; *p*-value < 2.2 × 10^−16^) (Figure 1e). Most of the putative lincRNAs had two exons. Fragments per kilobase of transcript per million (FPKM) analysis results showed that the expression level of putative lincRNAs was lower than protein-coding gene (mean value 0.77 vs. 31.57, respectively; *p*-value = 9.508 × 10^−13^) (Figure 1f). After converting genome coordinates, we found that 3.7% and 0.56% of exonic regions of putative lincRNAs in *Bos taurus* have orthologous regions in *Homo sapiens* and *Mus musculus*. And, 10.6% and 5.6% of exonic regions of protein-coding genes in Bos taurus have orthologous regions in *Homo sapiens* and *Mus musculus.* It can be implied that lincRNAs showed lower evolutionary conservation than protein-coding transcripts both in *Homo sapiens* and *Mus musculus* (Figure 1g). Taken together, these putative lincRNAs showed shorter length, less exon number, lower expression and evolutionary conservation than protein-coding transcripts.

**Figure 2:**
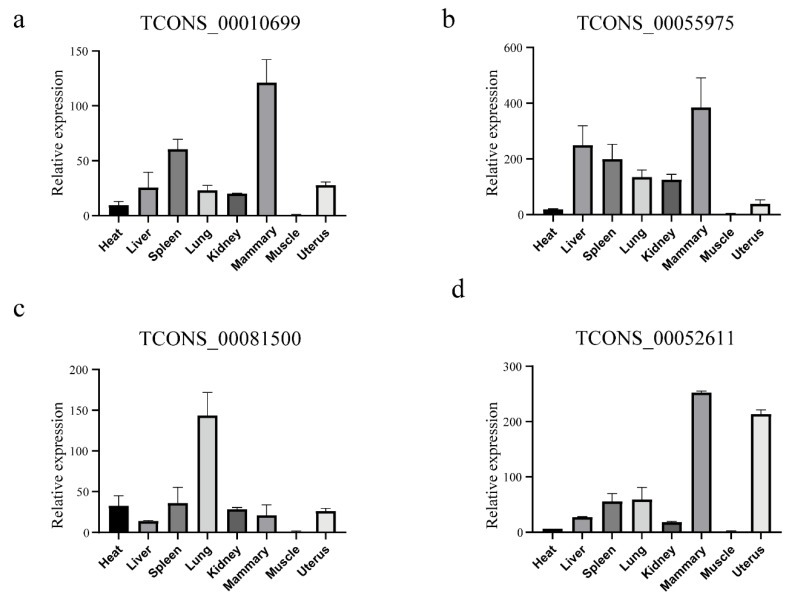
(**a**–**d**) Expression profile of four putative long intergenic non-coding RNAs (lincRNAs) in eight different tissues. Y axis represents relative expression level. Results are presented as mean values ± Standard error (SEM).

Previous studies showed that lincRNAs were remarkably tissue-specific than mRNA [32]. To confirm these identified lincRNAs, we randomly picked four novel lincRNAs to compare the expression level in mammary gland and other tissues by using the quantitative reverse transcription polymerase chain reaction (QRT-PCR). The results showed that these lincRNAs were highly expressed in mammary gland (Figure 2a–d).

### 3.2. Expression Analysis and Functional Prediction of DELs in MFP

**Figure 3:**
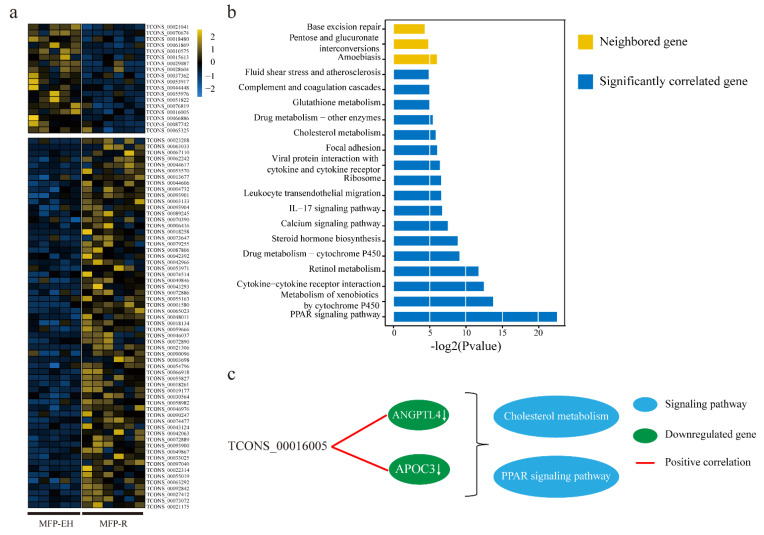
Functional analysis of differentially expressed lincRNAs (DELs) in mammary fat pad (MFP) under enhanced milk replacer (EH) vs restricted milk replacer (R). (**a**)The heat map of DELs in MFP under EH vs R. (**b**) The pathway analysis of DELs’ neighboring genes and significantly correlated genes (yellow, represent DELs’ neighboring genes; blue, represent significantly correlated differentially expressed genes (DEGs)) in MFP. (**c**) The selected DELs highly correlated with DEGs. (Arrow represent these genes were involved in those pathways)

A total of 79 DELs (61 upregulated, and 18 downregulated; EH vs R) were identified in response to different energy levels in MFP by using the unpaired *t*-test (Figure 3a). LincRNAs have been found to act as *cis-*element to participate in the transcriptional regulation of neighboring genes (<100 KB) [33,34]. According to the position of the genome, 261 genes (including 217 protein-coding genes) were found near those DELs (Supplement Table S4). These genes were involved in glucose metabolism and DNA repair signaling pathway (Figure 3b, upper yellow panel). Three of these nearby genes were also identified as DEGs in MFP (Table 1).

Besides, lncRNA could also act as trans-elements to regulate distant genes [35,36]. Those genes having similar co-expression patterns could be used to predict the putative lincRNA function. Therefore, we calculated the correlation between DELs and DEGs in MFP by using the Pearson correlation coefficient method (Supplement Table S5). A total of 488 DEGs (including 15 transcription factors) were found to be negatively or positively correlated with these 79 differentially expressed lincRNAs (*p*-value < 0.05). These DEGs were enriched in metabolism and signal transmission-related signaling pathways (Figure 3b, lower blue panel). Among them, peroxisome proliferators-activated receptors (PPARs) signaling pathway having the highest log_2_(*p*-value). Further analysis matched these DEGs with DELs to a total of 11,536 pairs. For example, TCONS_00016005 was positively correlated with apolipoprotein C3 (APOC3) and angiopoietin-related protein 4 (ANGPTL4), which were both enriched in PPAR signaling pathway and cholesterol metabolism pathway (Figure 3c).

### 3.3. Expression Analysis and Functional Prediction of DELs in PAR

**Figure 4:**
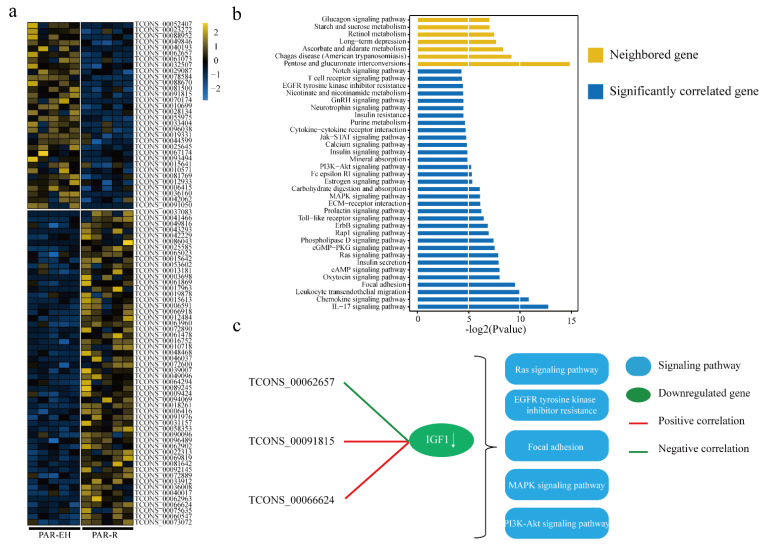
Functional analysis of differentially expressed lincRNAs (DELs) in mammary parenchyma (PAR) under enhanced milk replacer (EH) vs restricted milk replacer (R). (**a**) Heat map of DELs in PAR under EH vs R. (**b**) Pathway analysis of DELs’ neighboring genes and significantly correlated genes (yellow, represent DELs’ neighboring genes; blue, represent significantly correlated genes) in PAR. (**c**) Selected DELs that are highly correlated with DEGs. (Arrow represent the genes were involved in those pathways)

By using the same method, 86 differentially expressed lincRNAs (54 upregulated, 32 downregulated; EH vs R) in mammary parenchyma (PAR) were identified (Figure 4a). There were 281 genes (including 256 protein-coding genes) near these DELs (Supplement Table S4), including 19 DEGs (Table 1). These neighboring genes were mainly enriched in various metabolism pathways (Figure 4b, upper yellow panel). A total of 1453 DEGs (including 92 transcription factors) were found to be negatively or positively correlated with these 86 differentially expressed lincRNAs (Supplement Table S5). These DEGs were enriched in immune, cell proliferation, cell cycle and signal transduction pathways (Figure 4b, lower blue panel). Further analysis matched these DEGs with DELs to a total of 47,099 pairs. For example, TCONS_0062567 and TCONS_0091815 were positively and TCONS_0066624 was negatively correlated with Insulin Like Growth Factor I (IGF-I), which were enriched in MAPK signaling pathway, PI3K-Akt signaling pathway, Focal adhesion, Ras signaling pathway and epidermal growth factor receptor (EGFR) tyrosine kinase inhibitor resistance pathway and involved in the mammary gland development.

## 4. Discussion

Some studies showed that feeding heifer claves with restricted milk replacer was the right strategy to save rearing cost and increase milk production [37,38]. With the development of research, it has been confirmed that increased nutrient supply would benefit the development of mammary gland ultimately leading towards increased milk yield [39,40,41]. Furthermore, nutrient supply can affect the total protein, total DNA, total fat, protein and fat concentration changes in MFP and PAR. In order to reveal this molecular mechanism, a number of transcriptomic studies have been reported [42,43]. However, it is still largely unknown. Over the last decade, more and more studies showed that lncRNAs were the key regulators in various biological processes, and most of them were lincRNAs [10]. Although, PINC, NEAT1 and ZFAS1 were reported to be involved in mammary epithelial cell proliferation. Which lincRNAs are involved in the mammary gland development under different feeding regimes remained largely unknown. 

In this study, we have identified 534 putative lincRNAs from 22 samples from two tissues (MFP and PAR) and two treatments (EH, 1.13 kg/day, including 28% crude protein, 25% fat; R, 0.45 kg/day, including 20% crude protein, 20% fat), by using published high throughput *RNA*-seq data [18]. Results of characterization for these putative lincRNAs in our study are consistent with previous reports [44,45] which were also confirmed by QRT-PCR and it was concluded that most of the putative lincRNAs were highly expressed in mammary tissues.

In MFP tissue, those DELs neighboring genes and significantly correlated DEGs were enriched in various metabolism and signal transmission processes. Such as, PPAR signaling pathway, Calcium signaling pathway and Cytokine-cytokine receptor interaction, etc. Especially, the PPARs signaling pathway played a key function in preadipocyte proliferation [38,39]. In addition, we also found that these neighboring genes and significantly correlated DEGs were associated with lipid metabolism, which played a functional role in adipocyte tissue like, angiopoietin-related protein 4 (ANGPTL4), apolipoprotein C3 (APOC3), etc. [46,47]. And, these DEGs were downregulated in MFP under the restricted supply. Therefore, we speculated that these DELs might regulate neighboring genes and are significantly correlated with DEGs to promote MFP development under the enhanced nutrients supply. 

In PAR tissue, those DELs’ neighboring genes and significantly correlated DEGs were enriched in metabolism-related and cell proliferation-related pathways. Such as, PI3K-Akt signaling pathway, JAK-STAT signaling pathway and Ras signaling pathway, etc. Moreover, TCONS_0062567 was negatively correlated with Insulin Like Growth Factor I (IGF-I), while, TCONS_0091815 and TCONS_0066624 were positively correlated with IGF-I, which were considered as the important regulators in the lactation process [48]. Some other significantly correlated DEGs play indispensable role in mammary gland development like, Epidermal Growth Factor Receptor (EGFR) and Cyclin D2 (CCND2), etc. [49,50]. And, these DEGs were downregulated in PAR under the R supply suggesting that these DELs may promote the PAR development under the EH supply.

Mammary gland development is a complex process, including various tissues and cell types [1,51,52]. Both MFP and PAR play important roles in various stages of mammary gland development. In this study, only lincRNAs with ploy A tail were identified for this biological process. Functional prediction was accomplished for DELs highly correlated DEGs. However, further research can be conducted to explore specific functions and regulated mechanisms for these lincRNAs.

## 5. Conclusions

In summary, we identified 79 and 86 DELs in MFP and PAR of pre-weaning heifer calves under the enhanced and restricted nutrient supply. These putative lincRNAs may influence metabolism, cell proliferation and tissue interaction in MFP and PAR by positive or negative regulation to promote the mammary gland development and tissue communication under the enhanced nutrition supply. In this study, only those putative lincRNAs were studied which were highly correlated with these key protein-coding genes in mammary gland development. Moreover, it is suggested to assess the functional analysis of these putative lincRNAs by experiment.

## Figures and Tables

**Table 1:** Differentially expressed lincRNAs’ (DELs’) neighbored DEGs in PAR and MFP.

DELs ID	Neighboring Gene Name	Change (EH vs R)	Tissue
TCONS_00049867	ACVR2B	up	MFP
TCONS_00055163	LAMA1	down	MFP
TCONS_00027412	LOC100847119	up	MFP
TCONS_00063960	PYGM	up	PAR
TCONS_00033404	HRC	up	PAR
TCONS_00043293	THEMIS2	down	PAR
TCONS_00040017	LIMS2	up	PAR
TCONS_00049846	STAC	up	PAR
TCONS_00091815	PDE10A	down	PAR
TCONS_00016752	HCK	down	PAR
TCONS_00090096	NTRK2	down	PAR
TCONS_00039007	MRC2	down	PAR
TCONS_00033404	KCNA7	down	PAR
TCONS_00015641	PTGIS	up	PAR
TCONS_00015642	PTGIS	up	PAR
TCONS_00042229	SCN7A	up	PAR
TCONS_00036160	ACE	up	PAR
TCONS_00033404	LHB	down	PAR
TCONS_00040017	MYO7B	up	PAR
TCONS_00072600	GIMAP1	down	PAR
TCONS_00036008	PYY	up	PAR
TCONS_00033404	NTF4	down	PAR

## Data Availability

Data analyzed in this study were a re-analysis of published RNA-seq data, which are openly available at locations cited in the reference section [18]. Further documentation about data processing is available at [NCBI GEO DataSets] at [GSE102435].

## Supplementary Materials

The following are available online at https://www.mdpi.com/article/10.3390/ani11051268/s1, Table S1: Primers for real-time PCR assay used in this study, Table S2: The number of reads and mapping ratio of each sample, Table S3: The information of putative lincRNAs, Table S4: The alignment result of putative lincRNAs, Table S5: The correlation between DELs and DEGs.
